# Flexible expansive tubular endoscopic (Flextube) spine surgery for lumbar disc herniation: a case series of 35 patients with 6-month follow-up

**DOI:** 10.3389/fsurg.2026.1939957

**Published:** 2026-10-07

**Authors:** Zhipeng Wang, Bin Zhang, Zhishen Niu, Qi Wang, Kaiqiang Sun, Yuanzhen Li, Jiangang Shi, Jingchuan Sun

**Affiliations:** 1 Department of Orthopedic Surgery, Changzheng Hospital, Navy Medical University, Shanghai, China; 2 Department of Orthopedic, Affiliated Hospital of Gansu University of Traditional Chinese Midecine, Lanzhou, Gansu, China

**Keywords:** dual-medium endoscopy, endoscopic discectomy, flexible expansive tubular endoscopic, flextube, lumbar disc herniation, minimally invasive spine surgery

## Abstract

**Purpose:**

This study aimed to investigate the safety, efficacy, and clinical outcomes of Flexible Expansive Tubular Endoscopic (Flextube) spine surgery—a novel, non-rigid, air-medium endoscopic technique—for the treatment of single-level lumbar disc herniation (LDH).

**Methods:**

A retrospective case series was conducted on 35 consecutive patients with single-level LDH treated with Flextube surgery between January 2025 and June 2025. All procedures were performed using a flexible, atraumatic, expandable working tube. Clinical outcomes were evaluated preoperatively and at 1, 3, and 6 months postoperatively using the Visual Analogue Scale (VAS) for back and leg pain, Oswestry Disability Index (ODI), and modified MacNab criteria. Perioperative parameters including operative time, blood loss, length of hospital stay, and complications were analyzed.

**Results:**

A total of 35 consecutive patients (mean age 47.2 ± 12.3 years) with single-level lumbar disc herniation underwent Flextube surgery. All patients completed the 6-month follow-up. The mean operative time was 49.3 ± 11.7 min, and mean intraoperative blood loss was 14.8 ± 6.5 mL. The mean hospital stay was 1.7 ± 0.6 days. Clinical outcomes improved significantly at each postoperative follow-up compared with baseline. VAS back, VAS leg, and ODI scores improved significantly at each follow-up time point (*P* < 0.05). At 6-month follow-up, the excellent-good rate was 94.3% (33/35). No major complications such as dural tear, nerve root injury, or surgical site infection occurred.

**Conclusion:**

Flextube spine surgery is a safe and feasible minimally invasive procedure for single-level LDH, with satisfactory short-term clinical outcomes and a favorable safety profile. Further studies are needed to confirm its long-term efficacy and comparative advantages.

## 1 Introduction

Minimally invasive spine surgery (MISS) has become the mainstream treatment for lumbar disc herniation (LDH) over the past three decades. Compared with conventional open surgery, MISS is associated with reduced paraspinal muscle injury, less blood loss, shorter hospital stays, and faster functional recovery (1, 2). Currently, widely used MISS techniques include rigid tubular microendoscopic discectomy (MED), percutaneous endoscopic lumbar discectomy (PELD), and unilateral biportal endoscopy (UBE). However, these established techniques have inherent limitations. Rigid tubular retractors exert continuous, concentrated pressure on paraspinal muscles, potentially causing ischemia, reperfusion injury, and chronic postoperative back pain. Full-endoscopic and biportal techniques require continuous hypotonic fluid irrigation for visualization, which may lead to epidural hypertension, nerve root edema, unclear visual field, and even acute neurological deterioration (3, 4). Furthermore, these systems rely on expensive, specialized instruments and steep learning curves, limiting their widespread application in primary and secondary hospitals (5).

To overcome these drawbacks, we developed a novel surgical strategy: Flexible Expansive Tubular Endoscopic (Flextube) Spine Surgery. The core design of the Flextube system is a soft, flexible, gradually expandable working channel that conforms to anatomical curvature without rigid muscle retraction. The entire procedure is performed in an air-medium environment without fluid irrigation, providing clear, stable visualization. The system is compatible with standard arthroscopic or endoscopic equipment, greatly reducing costs and technical barriers.

To date, no clinical studies have reported the safety and efficacy of the Flextube technique for LDH. Therefore, this study presents a 6-month follow-up case series of 35 consecutive patients to evaluate the clinical outcomes, safety profile, and perioperative characteristics of this novel technique.

## 2 Materials and methods

### 2.1 Statement of ethics

All procedures involving human participants were in accordance with the ethical standards of the institutional research committee [IRB No. (2025-033)]. Informed consent was obtained from all individual participants included in the study.

### 2.2 Study design and population

This was a retrospective, single-center, consecutive case study performed between January 2025 and June 2025. Patients with single-level LDH refractory to conservative treatment for at least 6 weeks and treated with Flextube surgery were enrolled.

Inclusion criteria:

(1) Age 18–70 years;
(2) Single-level LDH confirmed by magnetic resonance imaging (MRI);
(3) Radicular pain consistent with imaging findings;
(4) Failed conservative treatment including physical therapy, analgesics, and epidural steroid injection;
(5) Complete follow-up data for at least 6 months.

Exclusion criteria:

(1) Lumbar spinal stenosis, spondylolisthesis > grade I, scoliosis > 10°;
(2) Multi-level disc herniation;
(3) Revision surgery, spinal tumor, infection, or fracture;
(4) Severe medical comorbidities precluding minimally invasive surgery.

### 2.3 Surgical technique

All procedures were performed by two senior spine surgeons using the Flextube system under general anesthesia. The surgical schematic illustration of Flextube surgery was shown in Figure 1.

**Figure 1:**
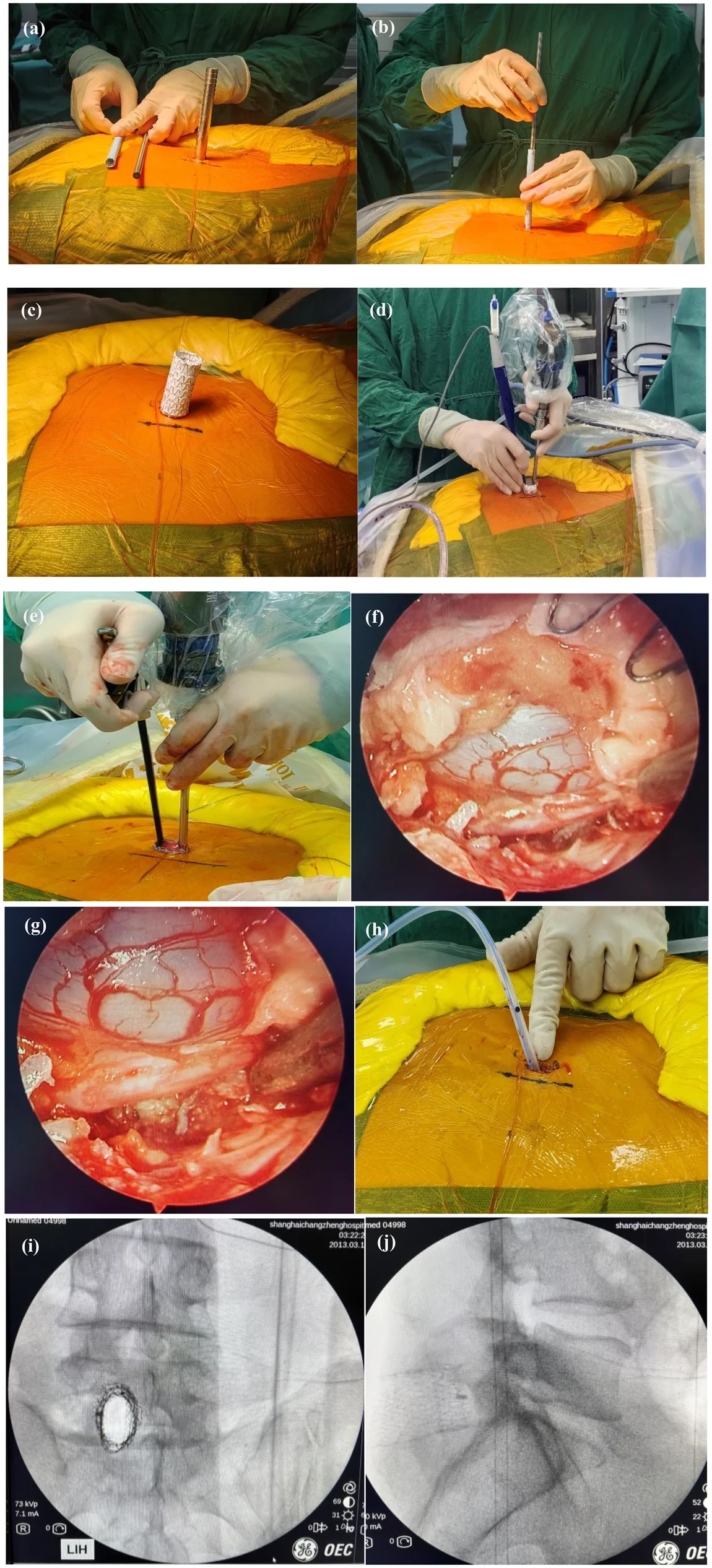
Surgical schematic illustration of flextube surgery. **(a–c)** Dilatation and placement of the flexible expansive tube. **(d,e)** Endoscopic view inside the Flextube under air medium. **(f)** The surgical field of the flexible expansive tube under the air medium. **(g)** Final decompression confirmed. **(h)** Wound closure with a small incision (∼1.5 cm). **(i,j)** x-ray fluoroscopy confirms the position of the channel.

#### 2.3.1 Patient positioning and localization

Following the induction of general anesthesia and confirmation of adequate depth of anesthesia, the patient was placed prone on the radiolucent operating table with the abdomen suspended to reduce intra-abdominal pressure and reduce intraoperative bleeding. The surgeon initially located the operative level by the anatomical landmarks on the surface of the body, and then under the guidance of the C-arm x-ray machine, the Kirschner wire was used to accurately identify the involved intervertebral space. After the C-arm fluoroscopy, the skin incision location was marked approximately 2.0 cm lateral to the midline.

#### 2.3.2 Soft-tissue dissection and Flextube placement

A 1.4–1.6 cm longitudinal skin incision was made in the skin directly to the deep fascia. The thoracolumbar fascia was bluntly separated without electrotomy, so that the subcutaneous tissue could be avoided from electrotomy as much as possible, which was conducive to better wound healing. The Flextube (Jiangsu Yuanmai Medical Equipment Technology Co., Ltd., China) —a flexible, non-irritating expandable tubular device—was inserted along the natural muscle space. The tube was gradually expanded to 18–22 mm to form a stable, atraumatic working channel. This procedure does not require the use of radiofrequency ablation, and the surrounding soft tissue and muscle in the surgical field can be completely blocked out of the working channel. The Flextube device, preloaded within its rigid introducer sheath, was gently advanced through the paraspinal muscle interval. Once the target level was confirmed fluoroscopically, the inner push rod was used to deploy the flexible tube distally. The distal end was carefully engaged with the dorsal cortex of the lamina and the facet joint complex, providing a stable and non-slip foundation for gradual tube expansion. This step effectively isolated the overlying musculature and maintained a clear trajectory for subsequent endoscopic visualization. Figures 1a–c illustrates this process.

#### 2.3.3 Dual-medium endoscopic discectomy

After the working channel was established, a standard 4-mm 30° arthroscope/endoscope was inserted into the Flextube. The light source was connected, revealing the articular capsule of the articular process. Common bipolar coagulation was used for local hemostasis, the joint capsule and upper and lower lamina Windows were exposed, and the surface soft tissue was cleaned with nucleus pulposus forceps. The initial dissection and partial resection of the ligamentum flavum were performed in an air medium without continuous fluid irrigation. A high-speed drill was used to remove part of the bone of the superior articular process, shape the articular process, and enlarge the intervertebral foramen. After the epidural space was entered, the surgical medium was smoothly converted to a water medium for enhanced visualization and hemostasis. The field of view included the upper and lower articular processes, upper and lower laminae, ligamentum flavum, dural sac, nerve roots and intervertebral discs. The working channel can expose the herniated disc below the nerve root without repositioning. The nerve root was protected using a retractor, the annulus fibrosus was incised with blue forceps, and the herniated nucleus pulposus was removed with a nucleus pulposus forceps. The herniated disc tissue was identified and completely removed under clear dual-medium visualization. The exposed nerve roots were relaxed and without tension, indicating adequate decompression. The nerve root was thoroughly decompressed and protected during the operation without prolonged irrigation with continuous perfusate.

#### 2.3.4 Hemostasis and wound closure

Meticulous hemostasis was achieved using a low-temperature radiofrequency device. The Flextube was removed, and the wound was closed in layers without a drainage tube. Postoperative care was provided as routine.

### 2.4 Evaluation and follow-up

The following variables were used to assess the efficacy and safety of the surgery: Disease duration

(months); protrusion segments; operative time (minutes); length of hospital stay (days); estimated blood loss (mL); duration of follow-up; change in pre- to postoperative pain score, quantified using a visual analog scale for back pain (VAS Back, 0–10), VAS score for leg radicular pain (VAS Leg, 0–10 ) (6), and disability, quantified using the Oswestry Disability Index (ODI, 0%–100% ) (7); patient satisfaction, quantified using the MacNab scale (excellent, good, fair, or poor); rate of re-operation (%); incidence rate of dural tears, nerve root injury, epidural hematoma, deep infection, or visceral injury; and the radiological outcomes of treatment. Other complications, such as delayed wound healing, epidural hematoma, headache, and neck pain, are also rare complications. The follow-up time was 6 months after surgery.

### 2.5 Statistical analysis

Statistical analysis was performed using SPSS 26.0 (IBM corporation, Amonk, USA). Continuous data were expressed as mean ± standard deviation (SD). Preoperative and postoperative outcomes were compared using paired t-tests. A P-value < 0.05 was considered statistically significant.

## 3 Results

### 3.1 Baseline characteristics

Over the 6 months of the study period, 35 patients were enrolled, including 19 males and 16 females. Mean age was 47.2 ± 12.3 years (range 21–68 years). The distribution of levels was as follows: L3–4 (2 cases), L4–5 (22 cases), L5–S1 (11 cases). Mean body mass index (BMI) was 24.7 ± 3.1 kg/m^2^. The mean duration of disease was 6.12 ± 1.29 months.

### 3.2 Perioperative indicators

The mean operative time was 49.3 ± 11.7 min (range 36–78 min), and the mean estimated blood loss was 14.8 ± 6.5 mL (range 5–30 mL). The mean length of hospital stay was 1.7 ± 0.6 days (range 1–3 days).

### 3.3 Therapeutic efficacy rate

The modified MacNab criteria data of all patients at 6 months after operation was 94.3%. No conversions to open or rigid tubular surgery occurred.

### 3.4 Clinical outcomes

In this study, the postoperative VAS-Back and VAS-Leg score of patients decreased from 6.8/7.5 preoperatively to 1.4/1.1 at 6 months postoperatively, with a reduction of over 5 points. Moreover, the ODI score decreased by approximately 50.0 points. All clinical scores improved significantly and continuously at 1-, 3-, and 6-month follow-ups (Table 1). The line chart was shown in Figure 2.

**Table 1:** Preoperative and postoperative clinical outcomes (*n*, M ± SD).

Outcome	Preoperative	1 Month	3 Months	6 Months	*P*-value
VAS-Back	6.8 ± 1.3	2.1 ± 0.8	1.6 ± 0.7	1.4 ± 0.6	< 0.001
VAS-Leg	7.5 ± 1.2	1.8 ± 0.7	1.3 ± 0.6	1.1 ± 0.5	< 0.001
ODI	62.4 ± 8.6	22.7 ± 6.3	15.6 ± 5.2	12.8 ± 4.7	< 0.001

**Figure 2:**
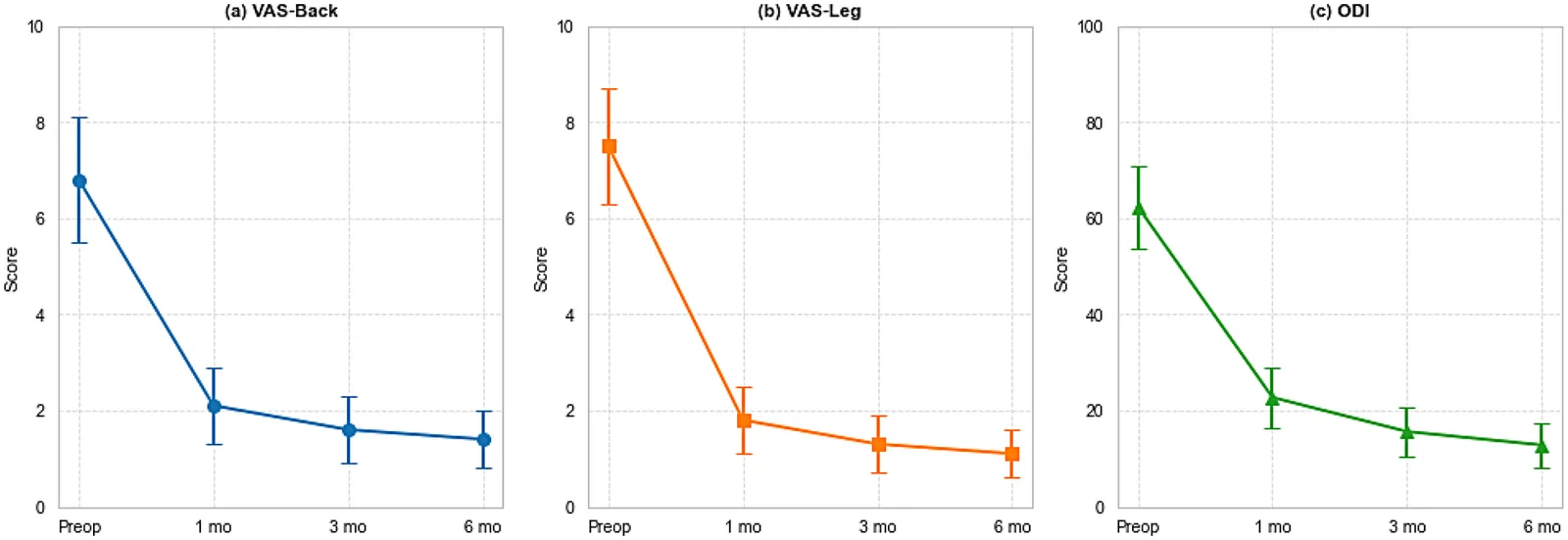
Line chart of VAS-back **(a)**, VAS-leg **(b)**, and ODI **(c)** over time. *X*-axis: Preop, 1 mo, 3 mo, 6 mo. *Y*-axis: Score. Three curves show continuous and significant improvement.

At 6-month follow-up, the modified MacNab criteria were show in Table 2. Preoperative and postoperative MRI of a representative case was shown in Figure 3.

**Table 2:** Modified macNab criteria data (n, %).

Outcome	Excellent	Good	Fair	Poor	Overall efficacy
MacNab	21 (60.0%)	12 (34.3%)	2 (5.7%)	0 (0.0%)	33 (94.3%)

**Figure 3:**
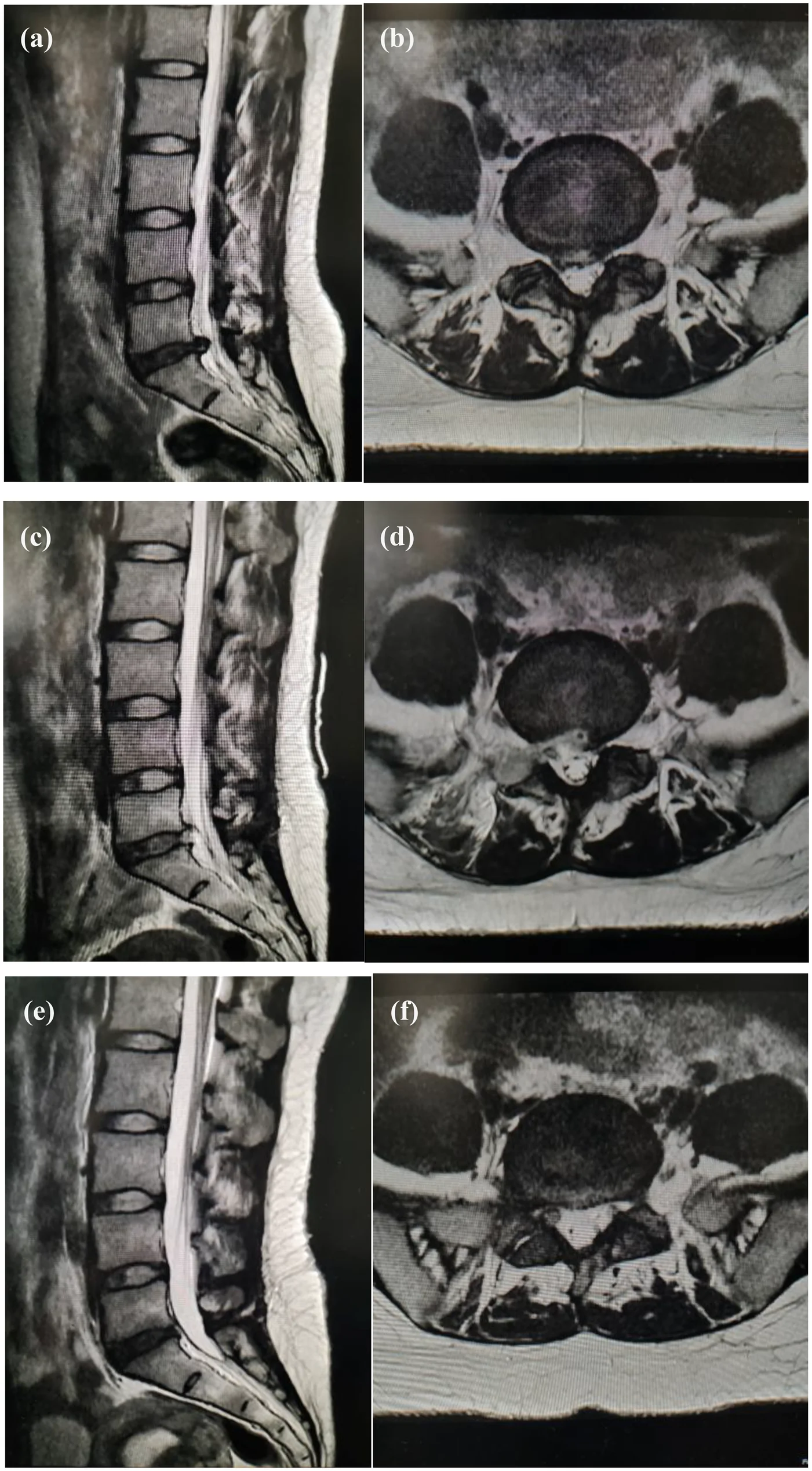
Preoperative and postoperative MRI of a representative case. **(a)** Preoperative sagittal T2WI: Severe L5-S1 disc herniation. **(b)** Preoperative axial T2WI: Significant nerve root compression. **(c,e)** Sagittal T2WI at 3 days and 6 months after surgery: Complete decompression. **(d,f)** Axial T2WI at 3 days and 6 months after surgery: Nerve root fully released.

### 3.5 Complications

Dural tear is a major complication of endoscopic spine surgery. Before resection of the ligamentum flavum during surgery, the dura and ligaments must be carefully and gently separated. No major complications occurred, including dural tear, nerve root injury, epidural hematoma, deep infection, delayed wound healing or visceral injury. Two patients reported transient local paresthesia, which resolved spontaneously within 2 weeks without intervention.

### 3.6 Advantages

The soft tissue, fascia and muscle tissue were isolated from the working channel after the soft channel was placed, and there was no need to use high-power radiofrequency ablation to make the cavity. The use of ordinary bipolar coagulation for hemostasis is simple and efficient. In the air medium, the anatomical identification was more clear in the surgical field, and it was not easy to get lost. After the anatomical structure is clearly identified, the superior articular process can be reconstructed under the air medium, and the ligamentum flavum and part of the lamina can be removed to expose the spinal canal. It can avoid the stimulation of nerve root and dural sac caused by long time irrigation of lavage fluid, and reduce the occurrence of paresis and dysesthesia after operation.

## 4 Discussion

Minimally invasive spinal surgery has become increasingly widespread for the treatment of lumbar disc herniation because it offers reduced soft-tissue injury, earlier mobilization, shorter hospital stays, and lower complication rates compared with conventional open surgery (8, 9). Numerous minimally invasive techniques have been established, including microendoscopic discectomy (MED), percutaneous endoscopic lumbar discectomy (PELD), unilateral biportal endoscopy (UBE), and arthroscopic-assisted uniportal spinal surgery (AUSS) (10–12). However, these techniques rely on rigid working channels, specialized instruments, or continuous fluid irrigation, which may lead to iatrogenic muscle injury, blurred visualization, or fluid-related complications (13). Continuous long-term pressure of the irrigation fluid may cause paresis and dysesthesia. To overcome these limitations, the present study introduced Flexible Expansive Tubular Endoscopic (Flextube) Spine Surgery and reported the clinical outcomes of 35 patients with 6-month follow-up. To the best of our knowledge, this is the first clinical case series investigating the safety and efficacy of the Flextube technique for lumbar disc herniation.

The most important finding of the present study is that Flextube surgery provides a safe, effective, and minimally invasive alternative for lumbar disc herniation. At the 6-month follow-up, the excellent and good rate reached 94.3%, which was comparable to that reported for conventional MED, PELD, and UBE (14, 15). Meanwhile, the procedure was associated with negligible blood loss, short operative time, minimal tissue trauma, and zero major complications. These results confirm that Flextube surgery is reliable and reproducible in clinical practice.

The core value of the Flextube flexible working channel is its deformable, flexible structure allows the channel itself to adapt dynamically to the arthroscope and instruments, thereby avoiding collision and interference between instruments and the endoscope within a narrow working space. In conventional rigid tubular systems such as MED/UBE, the fixed inner diameter and non-deformable wall restrict the movement of both the endoscope and instruments, frequently causing instrument clash and conflicting trajectories (16). This significantly limits surgical maneuverability, narrows the effective working space, and increases the technical difficulty of the procedure. In contrast, the flexible, adaptable Flextube can deform passively to accommodate different angles and paths of the arthroscope and instruments. This dynamic compliance eliminates instrument collision, expands the effective working area, and reduces the technical demands of the operation, making the procedure smoother and safer.

Compared with minimally invasive procedures such as UBE and AUESS, which largely abandon working channels, the use of a flexible soft channel provides better protection for the surrounding soft tissues. Specifically, during repeated in-and-out passage of surgical instruments, the Flextube prevents soft tissues from being repeatedly scraped, damaged, or entangled around the shaft of surgical devices. This advantage is particularly significant during annular suturing and other precise endoscopic procedures. The flexible channel maintains a clear and stable working pathway, shields soft tissues from interference, and thus improves the safety and efficiency of delicate operations (17).

A unique and clinically important characteristic of Flextube spine surgery is its ability to support dual-medium visualization—using either an air medium or water medium according to surgical requirements. This represents a major advantage over conventional endoscopic techniques, which rely on fixed fluid irrigation.

During the initial phase of surgical approach, including establishment of the working channel, dissection of the paraspinal muscles, and exposure and resection of the ligamentum flavum, the procedure can be safely performed under an air medium. This allows the use of electrocautery and bipolar coagulation rather than radiofrequency ablation, enabling faster and more efficient tissue dissection. Air-medium visualization provides a clear and stable surgical field during extraspinal and paraspinal phases, allowing rapid and precise exposure of the spinal canal.

Once the spinal canal is entered and neural structures are exposed, the surgical environment can be smoothly converted to water medium (18). Within the canal, numerous small epidural veins are prone to bleeding, and air-medium visualization may become unclear. Furthermore, because neural structures are already identified and protected, high-definition anatomical detail is less critical at this stage. Water-medium visualization significantly improves hemostasis, as bleeding from epidural veins can be efficiently controlled with radiofrequency (19).

Owing to the flexible and non-rigid nature of the Flextube, the pressure generated by water irrigation remains within a safe physiological range. Similar to the mechanism of AUSS (Arthroscopic Uniportal Spine Surgery), irrigation functions primarily to wash away blood and tissue debris rather than to achieve hemostasis by hydraulic pressure. Therefore, the procedure does not cause intraspinal hypertension or neurological complications.

This dual-medium strategy combines the efficiency of air-medium dissection outside the canal with the clarity and safety of water-medium visualization inside the canal (20). Switching between the two different media can solve the problem that the endoscope tip is easy to be contaminated by blood in the dry media environment. It provides both excellent visualization and high surgical safety, making Flextube surgery a reliable and minimally invasive approach for lumbar discectomy.

Furthermore, the Flextube system demonstrates excellent compatibility with standard arthroscopic or endoscopic equipment, which markedly improves clinical accessibility. MED and specialized spinal endoscopic systems require expensive, proprietary instruments and dedicated optical devices that are not universally available in primary or community hospitals (21). Conversely, Flextube surgery uses only a standard high-resolution arthroscope and conventional orthopedic instruments. The arthroscope provides clearer imaging and greater maneuverability than traditional dedicated spinal endoscopes. Because the equipment is widely available, the technique can beimplemented with minimal additional cost, making it particularly suitable for widespread application in low-resource settings.

Compared with specific established techniques, the Flextube surgery demonstrates several distinctive features. The Destandau technique (rigid tubular microendoscopic discectomy) relies on a fixed-diameter metal tube that exerts continuous pressure on paraspinal muscles, potentially leading to ischemia and postoperative back pain. In contrast, the flexible and expandable nature of the Flextube distributes pressure more evenly and reduces muscle compression. Compared with interlaminar monoportal endoscopy, which requires continuous fluid irrigation throughout the procedure, Flextube surgery allows dual-medium switching, thereby avoiding prolonged dural sac and nerve root exposure to irrigation pressure. Recently, Kaushal et al. described an arthrospine-assisted conversion to free-hand single-port saline endoscopy technique that also employs dual-medium visualization (22). However, their method requires conversion from a dry arthroscopic approach to a free-hand saline endoscopy, which may increase technical complexity and soft-tissue disruption. Flextube surgery, by contrast, maintains a consistent working channel throughout the procedure, allowing seamless medium switch without instrument or channel exchange, thus simplifying the surgical workflow and reducing operative time.

Several limitations of the present study should be acknowledged. First, this is a retrospective, single-center case series with a relatively small sample size, which may limit the generalizability of our findings. Second, the absence of a control group precludes direct comparative analysis of efficacy and safety. Third, the follow-up period was limited to 6 months; therefore, long-term outcomes regarding recurrence, adjacent segment degeneration, and functional maintenance remain unknown. Fourth, the selection criteria may have introduced bias, as only patients with single-level LDH and no significant comorbidities were included. Finally, the learning curve and cost-effectiveness were not formally evaluated in this study. Prospective, multicenter, randomized controlled trials with larger sample sizes and longer follow-up are warranted to validate the long-term efficacy and cost-effectiveness of Flextube surgery.

Based on this case series with 6-month follow-up, Flextube spine surgery appears to be a safe and feasible minimally invasive procedure for single-level lumbar disc herniation, with favorable short-term clinical outcomes, low complication rates, and rapid postoperative recovery. The dual-medium capability and flexible working channel offer potential advantages in terms of tissue protection and surgical visualization. The technique achieves satisfactory short-term clinical outcomes and may serve as a next-generation minimally invasive strategy for the treatment of lumbar disc herniation. However, claims regarding cost-effectiveness, learning curve, and long-term efficacy require further comparative and prospective studies with extended follow-up.

## Data Availability

The original contributions presented in the study are included in the article/Supplementary Material, further inquiries can be directed to the corresponding authors.
